# Association of Polymorphisms in Oxidative Stress Genes with Clinical Outcomes for Bladder Cancer Treated with Bacillus Calmette-Guérin

**DOI:** 10.1371/journal.pone.0038533

**Published:** 2012-06-12

**Authors:** Hua Wei, Ashish Kamat, Meng Chen, Hung-Lung Ke, David W. Chang, Jikai Yin, H. Barton Grossman, Colin P. Dinney, Xifeng Wu

**Affiliations:** 1 Department of Epidemiology, The University of Texas MD Anderson Cancer Center, Houston, Texas, United States of America; 2 Department of Urology, The University of Texas MD Anderson Cancer Center, Houston, Texas, United States of America; UCSF/VA Medical Center, United States of America

## Abstract

Genetic polymorphisms in oxidative stress pathway genes may contribute to carcinogenesis, disease recurrence, treatment response, and clinical outcomes. We applied a pathway-based approach to determine the effects of multiple single nucleotide polymorphisms (SNPs) within this pathway on clinical outcomes in non-muscle-invasive bladder cancer (NMIBC) patients treated with Bacillus Calmette-Guérin (BCG). We genotyped 276 SNPs in 38 genes and evaluated their associations with clinical outcomes in 421 NMIBC patients. Twenty-eight SNPs were associated with recurrence in the BCG-treated group (*P*<0.05). Six SNPs, including five in *NEIL2* gene from the overall and BCG group remained significantly associated with recurrence after multiple comparison adjustments (*q*<0.1). Cumulative unfavorable genotype analysis showed that the risk of recurrence increased with increasing number of unfavorable genotypes. In the analysis of risk factors associated with progression to disease, rs3890995 in *UNG*, remained significant after adjustment for multiple comparison (*q*<0.1). These results support the hypothesis that genetic variations in host oxidative stress genes in NMIBC patients may affect response to therapy with BCG.

## Introduction

In the United States, bladder cancer is the fourth most common cancer and the eighth leading cause of death from cancer in men, with an estimated 69,250 new cases and 14,990 deaths in 2011 [1]. Most (75%–85%) bladder cancers are non–muscle invasive at first diagnosis, which include superficial non-invasive (pTa), minimally-invasive (pT1), or carcinoma in situ (CIS) [2]. Overall bladder cancer recurrence rates of non–muscle invasive bladder cancer (NMIBC) have been reported to be 31–78% at 5 years, and for high risk groups, 17–45% patients will progress to muscle invasive disease at 5 years [3]–[4]. Transurethral resection (TUR) is the gold standard for diagnosis and low risk Ta T1 transitional cell carcinoma (TCC) treatment [5]. However, recurrence at 3 months for patient with multifocal tumors varied between 7% and 46%, suggesting different TUR qualities [5].

Tumor cells often show an increased level of DNA oxidation products and oxidation-induced mutations [6]. Oxidative stress causes protein, lipid, and DNA damage and is a critical pathophysiological event implicated in several human pathologies, including cancer [7]–[8]. However, accelerated repair of oxidized DNA can protect cancer cells from harmful oxidative DNA damage [9] and is associated with metastasis and poor patient survival [10]. Many oxidative stress genes, such as e*NOS, MnSOD,* and *GPX* are polymorphic and have been studied for association with cancer risks and clinical outcomes [11]–[14].

To reduce the recurrence and progression of NMIBC, intravesical installation of Bacillus Calmette-Guérin (BCG) is often administered after TUR. BCG administration has been reported to induce local oxidative stress, which may mediate the antitumor activity [15]. We hypothesized that polymorphisms of oxidative stress–pathway genes may modulate the risk of recurrence and progression in BCG treated patients. To test this hypothesis, we conducted a study to evaluate the effects of 276 single-nucleotide polymorphisms (SNPs) in 38 key genes from the oxidative-stress pathway. To our knowledge, this is the first study to explore the association between a comprehensive panel of polymorphisms of the oxidative stress–pathway genes and risk of recurrence or progression in NMIBC patients, particularly in those receiving BCG therapy.

## Materials and Methods

### Ethics Statement

Written informed consents were obtained from all patients prior to enrollment in this study. The study was approved by the Institutional Review Boards at MD Anderson Cancer Center and Baylor College of Medicine.

### Study Population

The study population and enrollment procedures were described previously [16]. Patients were recruited from The University of Texas MD Anderson Cancer Center and Baylor College of Medicine as a part of an ongoing project since 1999. All patients were identified by reviewing daily electronic clinic schedules at the Urology and GU Medical Oncology clinics. The NMIBC patients were newly diagnosed, histologically confirmed, and had not been previously treated with chemotherapy or radiotherapy at the time of recruitment. There were no restrictions for age, gender, ethnicity, histology or stage on recruitment. Since more than 90% of our recruited cases were transitional cell carcinoma (TCC), we only included TCC cases in this study. In addition, the majority of patients in our study population (90.6%) were Caucasians; therefore, we restricted the analysis to Caucasians to limit the confounding effect from population stratification. A total of 421 NMIBC patients were included in this study.

### Epidemiological and Clinical Data Collection

M.D. Anderson staff interviewers administered a 45-minute in-person interview to all participants to collect data on demographic characteristics, tobacco use history, medical history, occupation and exposure, and family history of cancer. At the end of the interview, all participants donated a small blood sample for DNA extraction before receiving any treatment. Patients treated with TUR were followed up with periodic cystoscopic examinations, and subsequently some were treated with BCG. This treatment consisted of either BCG for 6 weekly instillations (induction BCG, iBCG) or iBCG + maintenance BCG (mBCG) in 3 weekly cycles given at 3, 6, 12, 18, 24, and 30 months after iBCG [17]. Clinical data were obtained from medical records by trained chart review specialists. The end points of outcome assessment in this study included recurrence and progression, which was calculated from the date of diagnosis to the date of endpoint events, death or last followup, whichever came first. Tumor recurrence was defined as a newly detected bladder tumor following a negative follow-up cystoscopy, and tumor progression was defined as the transition from non–muscle-invasive to invasive or metastatic disease. Patients who died or lost to follow up before the endpoint events were censored.

### Selection and Genotyping of Polymorphisms in Oxidative Stress–Pathway Genes

We compiled gene list using SNPs3D bioinformatic tools (http://www.snps3d.org) [18]. We then identified tagging SNPs from the Hapmap database (http://www.hapmap.com). Similar to our previous study [19], all SNPs fit the following criteria: *r^2^*<0.8, minor allele frequency (MAF) >0.05, and located within 10 kb upstream of the 5′-untranslated region (UTR) and 10 kb downstream of the 3′-UTR of the gene. We also included potentially functional SNPs with MAFs >0.01 (e.g., coding SNPs and SNPs in UTRs, promoters, or splice sites).The genotyping was performed using Illumina’s iSelect custom SNP array (Illumina, San Diego, CA) and the average call rate was 99.7%.

### Luciferase Reporter Assay to Determine Effect of the NEIL2:rs4639 Alleles on Regulation of NEIL2 Expression by microRNAs

A reporter construct derived from pGL3 (Ambion) containing the 3′-UTR of *NEIL2* was generated as follows: a 1.35-kb fragment of the *NEIL2* 3′-UTR was amplified from genomic DNA with the following primers: Forward1, 5′-GAAGATGGGTTACAGAGGCT; Reverse1, 5′-CAGACATCGTGGTGACAGAG. Subsequently, a 0.35-kb nested PCR fragment of 3′-UTR containing rs4639 was generated with primers: Forward2, 5′- AATTCTAGAGGGATACAGGCACCAAGAGGCGG; Reverse2, 5′- GTAGGCCGGCCGCGAGTAACAGTGAGCTTTAT. The amplified product was digested with XbaI and FseI and subcloned into pGL3 control vector. Variant *NEIL2* allele construct was generated by site-directed mutagenesis with primers: Forward3, 5′- GATTTGTTGgACAATTCAGGAATCAAGG and Reverse3, 5′-TCCTGAATTGTcCAACAAATCAACAGTCAC.

UM-UC-9 bladder epithelial cancer cells were kindly provided by Dr. Barton Grossman’s lab and were authenticated within six months prior to our study [20]. UM-UC-9 cells were transfected with 0.5 µg of the wild-type or variant *NEIL2* 3′-UTR construct along with 5 pmol of negative RNA control (scrambled sequence), miR-421, or miR-1200 microRNA (miRNA) mimic (Sigma-Aldrich, St. Louis, MO) and 10 ng of pGL4 Renilla luciferase reporter (Ambion, Carlsbad, CA) using Lipofectamine 2000 (Invitrogen, Carlsbad, CA) for 36 hours. The reporter expression was analyzed using the Dual-Luciferase Reporter Assay System (Promega, Madison, WI) and luciferase activities were measured in a FLUOstar Optima microplate reader (BMG Labtech, Cary, NC). Each assay was repeated independently three times with five replicates.

### Statistical Analyses

Statistical analyses were performed using STATA 10.0 (Stata Corp., College Station, TX, USA). The χ^2^ test (for categorical variables) or Student’s *t*-test (for continuous variables) was used to compare characteristics between the groups of patients with or without disease outcome. Linkage disequilibrium (LD) between individual SNPs was evaluated, with *r^2^*>0.8 were defined as being in the same haplotype block.

For each patient, we calculated the recurrence-free survival time as the time from diagnosis to recurrence, progression or to the date of death or last follow-up. Patients who were lost to follow-up or died before the end-point event were censored. We calculated person-years at risk within each genotype category as the sum of the recurrence-free survival times of all subjects in that genotype category. The effects of genotypes of SNPs on bladder cancer outcomes were estimated as hazard ratios and 95% confidence intervals (95% CI) using multivariate Cox regression under the dominant, recessive, and additive models of inheritance adjusted for age, gender, and smoking status, where appropriate. To control for multiple comparison, a *q* value (a false discovery rate [FDR]-adjusted *P* value) was used to adjust the significance level for individual SNPs. The *q* value was calculated by the *q* value package implemented in the R software. A bootstrap resampling method, using 100 bootstrap samples, was applied to internally validate the results. *P* for trend value was calculated with the number of unfavorable genotypes as continuous variable using unconditional Cox regression.

All analyses were stratified by age, gender, smoking status, tumor stage, tumor grade, and treatment. For the cumulative effects of multiple genetic variants, the adverse genotypes were summed and categorized by the number of adverse genotypes in subjects. To evaluate the cumulative effects of genetic variants, we summed up adverse genotypes (genotypes associated with significantly increased risk in the main effect analysis after adjustment for multiple comparisons) for each subject. Kaplan-Meier plots and log-rank tests were applied to compare the differences between the recurrence-free survival times of different genotypes. Non-parametric Wilcoxon Ranksum test was used to compare differences in luciferase activities in the reporter assay. All statistical tests were two-sided.

## Results

### Characteristics of the Study Population

Of the 421 Caucasian patients we followed, 346 (82.2%) were men and 75 (17.8%) were women. Among them, 232 (55.1%) had disease recurrence, with a median recurrence-free survival time of 14.05 months. As shown in **Table S1,** no significant differences for recurrence rate were identified based on age (*P* = 0.68), sex (*P* = 0.11), smoking status (*P* = 0.69), cancer stage (*P* = 0.13), or cancer grade (*P* = 0.19). There were no significant differences in progression by age (*P* = 0.05) or smoking status (*P* = 0.96). Men had higher rates of disease progression than women (*P* = 0.01) and patients with a higher stage or grade were also more likely to experience progression (*P*<0.01). Since BCG was primarily administered to those with higher risk of recurrence [21], we compared different BCG treatments on risks of recurrence and progression. Compared to iBCG, mBCG treatment was significantly associated with reduced risks of recurrence and progression (*P*<0.01).

### Analyses of Disease Recurrence Stratified by Treatment

Twenty five SNPs were significantly associated with recurrence risk in overall patients, and after adjusting for multiple comparisons, 6 remained significant, with a *q*-value ≤0.1 (**Table S2**). In the TUR patients, 34 SNPs were significantly associated with recurrence risk (*P*<0.05) but after adjusting for multiple comparisons none remained significant (**Table S3**). In the BCG-treated patients, 28 SNPs were significantly associated with recurrence, and after adjusting for multiple comparisons, 6 remained significant, with a *q*-value ≤0.1 (**Table 1**; **Table S4**).

To internally validate these results, we performed random bootstrap sampling of the significant SNPs for 100 iterations and listed the number of times that the *P* value was <0.05. In BCG-treated patients, four of the five top SNPs in *NEIL2* had highly consistent results, with bootstrap *P* values <0.05 for greater than 90% of samplings (**Table 1**).

**Table 1:** Oxidative stress gene SNPs and disease recurrence in BCG-treated&, Texas NMIBC patients enrolled in an ongoing molecular epidemiologic study of bladder cancer, 1999-present.

				Recurrence in BCG subgroup					No. Times in bootstrap sample*P*<0.05
			Best Model#	Yes/No					
SNP*	Gene	Genotype		ww	wv	vv	HR (95%CI)†	*P*	
rs804256‡	*NEIL2*	T/C	RES	41/36	51/42	18/4	4.58(2.61–8.02)	1×10^−7^	91
rs804276‡	*NEIL2*	A/G	RES	33/31	47/41	30/10	2.71(1.75–4.20)	9×10^−6^	99
rs4639‡	*NEIL2*	A/G	RES	28/28	52/44	30/10	2.60(1.68–4.03)	2×10^−5^	98
rs2173962	*SOD1*	A/G	DOM	93/81	16/1	1/0	2.45(1.42–4.23)	1×10^−3^	58
rs804267‡	*NEIL2*	T/C	DOM	61/32	39/37	10/13	0.53(0.36–0.78)	1×10^−3^	92
rs8191604	*NEIL2*	A/C	DOM	73/40	31/39	6/3	0.54(0.36–0.81)	3×10^−3^	76

* SNPs that remained significant after controlling for multiple comparisons by *q*-value with FDR (false discovery rate) less than 10%. All *NEIL2* SNPs in this table were in different haplotype blocks with *r* ^2^<0.8.

† Adjusted by age, sex, smoking status, tumor stage, and tumor grade.

# Best model: the model with smallest *P* value; DOM: dominant model, RES: recessive model.

ww, homozygous wild-type genotype; wv, heterozygous variant genotype; vv, homozygous variant genotype.

‡ SNPs with *P* value <0.05 in least 80% of the bootstrap samples.

& Patients treated with iBCG or iBCG plus mBCG.

### Cumulative Effects of Multiple SNPs on Recurrence in NMIBC Patients

**Figure 1:**
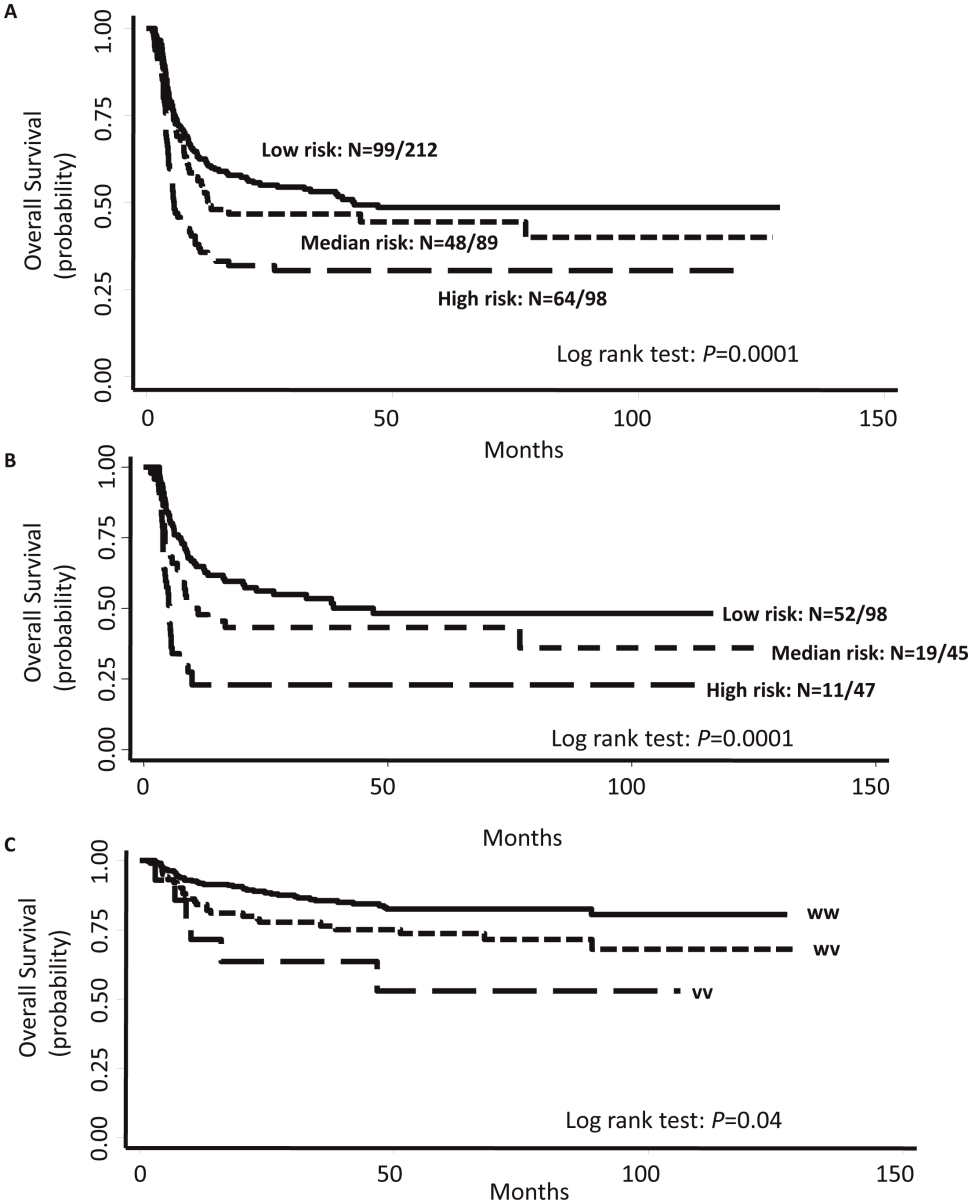
Kaplan-Meier estimates of recurrence-free or progression-free survival in NMIBC patients. (**A**) Kaplan-Meier estimates of recurrence-free survival in all NMIBC patients grouped by the three risk groups as categorized by the number of unfavorable genotypes in the oxidative stress pathway. Recurrence-free median survival time [MST]  = 42.3, 12.5, and 5.6 months for low, medium, and high risk patients, respectively (*P*_log-rank_ = 0.0001). (**B**) Kaplan-Meier estimates of recurrence-free survival in BCG-treated patients grouped by the three risk groups as categorized by the number of unfavorable genotypes in the oxidative stress pathway. MST  = 66.1, 25.4, and 16.6 months) for low, medium, and high risk patients, respectively (*P*_log-rank_ = 0.0001). (**C**) Overall Kaplan-Meier estimates of the effect of *UNG*:rs3890995 genotypes on progression-free survival in NMIBC patients (in the three risk subgroups, progression-free survival were greater than the follow-up time of 132.6 months). Compared with patients carrying homozygous variant genotype, those with at least one wildtype allele showed longer progression-free survival time (*P*_log-rank_ = 0.04). ww-homozygous wildtype; wv- heterozygous; vv-homozygous variant.

We summed the number of unfavorable genotypes for each individual and categorized the patients by tertile distribution of their recurrence risk, which showed dose-dependent trend among three groups (*P* trend <0.0001). In total patients, compared with the low-risk group, the median-risk and high-risk groups had a 1.2-fold (HR, 1.21, 95% CI, 0.85–1.71) and a 2.2-fold (HR, 2.21, 95% CI, 1.60–3.05) higher risk of recurrence, respectively (*P*<0.01; **Table 2**). Among BCG (iBCG alone or iBCG plus mBCG) treated patients, compared with the low-risk group, the median-risk and high-risk groups had a 1.46-fold (HR, 1.46, 95% CI, 0.9–2.38) and a 3.5-fold (HR, 3.52, 95% CI, 2.23–5.55) higher recurrence risk, respectively (*P*<0.01; **Table 2**). Kaplan-Meier estimates of the total and BCG-treatment groups indicated differences in median recurrence-free survival times (MST) (log rank test, *P* = 0.0001; **Figure 1 A** and **B**).

**Table 2:** Cumulative effect of unfavorable genotypes in oxidative stress pathway on total and BCG-treated NMIBC recurrence.

Total patients					BCG& treated patients				
*No. unfavorable genotypes	Yes (no.)	No (no.)	HR† (95% CI)	*P*	#No. unfavorable genotypes	Yes (no.)	No (no.)	HR† (95% CI)	*P*
0–1	99	113	Ref.		0–1	52	48	Ref.	
2	48	41	1.21 (0.85–1.71)	0.29	2	19	26	1.46 (0.90–2.38)	0.13
3–6	64	34	2.21 (1.60–3.05)	<0.01	3–6	11	36	3.52 (2.23–5.55)	<0.01
			*P* for trend <0.0001					*P* for trend <0.0001	

* Unfavorable genotypes in overall patients: *NEIL2* rs4639(vv); *NEIL2* rs804276(vv); *NEIL2* rs804256(vv); *NEIL2* rs1874546(vv); *NEIL2* rs804267(vv); *TDG* rs4135064(ww+wv).

# Unfavorable genotypes in BCG treated patients: *NEIL2* rs804256(vv); *NEIL2* rs804276(vv); *NEIL2* rs4639(vv); *SOD1* rs2173962(wv+vv); *NEIL2* rs804267(ww+wv); *NEIL2* rs8191604(ww+wv).

& Patients treated with iBCG or iBCG plus mBCG.

† HR: hazard ratio, CI: confidential interval. Adjusted by age, sex, smoking status, tumor stage, and tumor grade.

### Effects of SNPs on Disease Progression

In the NMIBC patients, 23 SNPs were significantly associated with progression (*P*<0.05, **Table** **3**). After adjusting for multiple comparisons, one SNP, *UNG*:rs3890995, remained significant (*Q* = 0.08) and had a highly consistent result, with bootstrap *P* value <0.05 for 90% times of samplings (**Table 3**). The median progression-free survival times were longer than the follow-up time of 132.6 months for all genotypes (**Figure 1 C**). However, patients who carried the homozygous variant alleles had a 1.92-fold (HR 1.92, 95% CI, 1.33–2.77) higher progression risk (**Table 3**) and had decreased progression-free survival (log-rank test, *P* = 0.04; **Figure 1** C). Due to the limited number of progression cases, we did not perform similar analysis in BCG-treated patients.

**Table 3:** Oxidative stress gene SNPs and disease progression in NMIBC patients receiving BCG treatment.

			Progression Yes/No					No. of bootstrap samples‡
SNP	Gene	Best Model#	ww	wv+vv	vv	HR (95% CI)†	*P*	*P*<0.05
rs3890995*	*UNG*	ADD	42/242	27/77	6/8	1.92(1.33–2.77)	5×10^−4^	90
rs1874546	*NEIL2*	DOM	34/201	35/100	5/24	1.89(1.18–3.03)	0.01	–
rs12674710	*NAT2*	REC	48/230	22/90	5/7	3.47(1.37–8.81)	0.01	–
rs11773597	*CYP3A4*	DOM	59/277	16/47	0/3	2.18(1.21–3.93)	0.01	–
rs1805410	*PARP1*	DOM	48/248	24/72	3/7	1.92(1.17–3.12)	0.01	–
rs1799930	*NAT2*	DOM	48/155	22/137	5/34	0.53(0.33–0.86)	0.01	–
rs1013358	*MPG*	ADD	49/254	22/68	4/5	1.67(1.13–2.48)	0.01	–
rs5751222	*CYP2D6*	REC	47/202	21/106	7/17	2.74(1.20–6.24)	0.02	–
rs9332197	*CYP2C9*	DOM	63/297	12/30	0/0	2.16(1.14–4.09)	0.02	–
rs3136717	*IKBKB*	DOM	49/259	26/64	0/0	1.80(1.10–2.92)	0.02	–
rs915908	*CYP2E1*	DOM	61/225	14/95	0/7	0.50(0.28–0.89)	0.02	–
rs1050112	*PARP4*	REC	25/136	36/151	14/40	2.00(1.10–3.65)	0.02	–
rs2475377	*CYP2C9*	DOM	65/305	10/22	0/0	2.24(1.12–4.49)	0.02	–
rs976072	*POLG*	REC	33/144	38/131	4/52	0.31(0.11–0.86)	0.02	–
rs1062492	*NUDT1*	DOM	43/217	31/95	1/15	1.71(1.07–2.74)	0.02	–
rs1858923	*ABCB1*	DOM	20/120	33/143	22/64	1.79(1.07–2.99)	0.03	–
rs1961456	*NAT2*	ADD	33/175	34/131	8/21	1.49(1.04–2.15)	0.03	–
rs4796030	*LIG3*	REC	24/96	42/158	9/73	0.46(0.23–0.94)	0.03	–
rs4680	*COMT*	DOM	52/188	19/119	4/20	0.60(0.36–0.98)	0.04	–
rs4633	*COMT*	DOM	24/72	34/180	17/72	0.60(0.37–0.98)	0.04	–
rs625978	*GSTP1*	DOM	26/73	32/162	17/92	0.61(0.37–0.99)	0.04	–
rs1866074	*TDG*	REC	16/81	51/174	8/72	0.47(0.22–0.98)	0.04	–
rs1045642	*ABCB1*	ADD	24/91	36/153	15/83	0.73(0.53–1.00)	0.05	–

* SNPs that remained significant after controlling for multiple comparisons by *q*-value. (FDR<10%).

# Best model: the model with smallest *P* value; ADD: additive model, DOM: dominant model, RES: recessive model.

† HR: hazard ratio, CI: confidential interval. Adjusted by age, sex, smoking status, tumor stage, tumor grade, and treatment.

‡ SNPs not significant by *q*-value were not tested in bootstrapping method.

### Effects of hsa-mir-421 and hsa-mir-1200 on NEIL2 Expression

**Figure 2:**
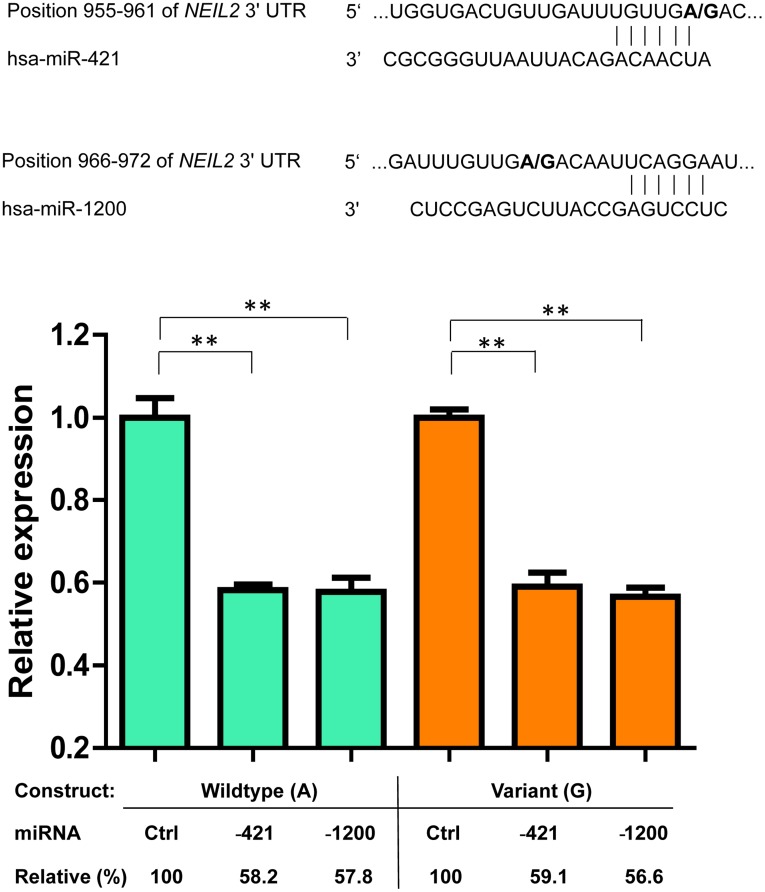
*NEIL2*:rs4639 alleles are targeted by miRNAs. rs4639 (A/G) SNP position and presumed pattern of miRNA binding with hsa-miR-421 and hsa-miR-1200 (Targetscan). Result of dual luciferase reporter assay showing the effects of rs4639 variant, hsa-mir-421 (−421), and hsa-mir-1200 (−1200) on reporter expression. All values were normalized to the *Renilla* luciferase activities and expressed as percentages of the luciferase activity for cells transfected with wild-type reporter construct and negative control RNA (Ctrl). Values are averages of five replicates with error bars for standard deviation. **, *P*<0.01 by Wilcoxon Ranksum test. Assay was repeated three times with similar results.

Since one of the most significant SNPs, *NEIL2*:rs4639, is located in the gene’s 3′UTR, we scanned the *NEIL2* 3′ UTR region for potential miRNA binding sites Targetscan (www.targetscan.org). Results showed that rs4639 allele is located close to a potentially weak binding site for the miRNAs hsa-miR-421 and hsa-miR-1200 (**Figure 2**). We cloned 3′UTR of *NEIL2* and analyzed the effects of this SNP on the miRNA-mediated expression regulation. The two miRNAs had similar inhibitory effects on both wildtype and variant rs4639 expression (**Figure 2**), suggesting *NEIL2*:rs4639 might not affect targeting by these miRNAs.

## Discussion

We evaluated the association of a panel of 276 SNPs in 38 oxidative-stress genes with the recurrence of NMIBC in patients treated with or without BCG. We identified 25 SNPs that were significantly associated with cancer recurrence in all (TUR- and BCG-treated) patients, 34 significant SNPs in TUR only patients, and 28 significant SNPs in BCG-treated patients. In addition, we found significant gene-dosage effects from the cumulative analysis, and identified one SNP associated with NMIBC progression.

The most significant SNP associated with recurrence in the BCG-treated group was *NEIL2:*rs804256, which is located in the intron region. Compared with the wild-type genotypes, the recurrence risk for the homozygous variant CC genotype was increased more than four-fold. However, this SNP was not significant in TUR only subgroup, suggesting a potential interaction of rs804256 with BCG treatment. Five out of six of the most significant SNPs are located in *NEIL2*, which highlights the importance of this gene in the recurrence risk. *NEIL2* belongs to a class of DNA glycosylases homologous to bacterial Fpg/Nei family, which is essential for removing cytosine-derived lesions, particularly 5-hydroxyuracil and 5-hydroxycytosine [22]. It has been reported that two 5′-UTR polymorphisms decrease *NEIL2* expression and increase mutagen-induced genetic DNA damage [23]. Downregulation of *NEIL2* expression may lead to increased oxidative stress and genomic instability in rapidly proliferating tissues [24]. *NEIL2* may also play important roles in repairing DNA damage induced by carcinogenic metals [25]. One of the most significant SNPs associated with recurrence, rs4639, is located in 3′-UTR of *NEIL2*. Luciferase reporter assay results showed that hsa-mir-421 and hsa-mir-1200 miRNAs had similar inhibitory effects on both wildtype and variant rs4639 expression. Since either miRNAs showed similar inhibitory effects on both wildtype and variant genotypes, it is possible that rs4639 may not be the causative variant but functions as a tagging SNP for other polymorphisms that may contribute to bladder cancer recurrence by altering *NEIL2* expression or function. Alternatively, the variant allele of this SNP may affect targeting by other miRNAs. Future fine mapping of the regions surrounding the most significant tagSNPs are needed to identify the causal genetic variations and their molecular mechanisms.

We also identified a significant gene-dosage effect for the six tagging SNPs that showed significant main effects. Patients with the largest number of unfavorable genotypes had the highest risk of NMIBC recurrence, suggesting that additional risk genotypes within this key pathway were detrimental. This highlights the importance of assessing multiple SNPs within a shared pathway for clinical outcome assessment.

One polymorphism, *UNG*: rs3890995, was significantly associated with NMIBC progression. Rs3890995 is located 1.87 kb upstream of *UNG* 5′ UTR. One important function of UNG is to recognize and remove uracil from DNA by cleaving the N-glycosylic bond and initiating the base-excision repair pathway. DNA uracil comes from cytosine deamination or misincorporation of dUMP residues [26]. It is possible that the variant allele of SNP rs3890995 may affect gene transcription thus altering protein level. Alternatively, it may be linked to other causal variants in *UNG*.

In summary, we showed that genetic polymorphisms of the oxidative stress pathway genes may modulate the risk of NMIBC recurrence and progression in BCG treated patients. Further, we have conducted a relatively comprehensive query of the oxidative stress pathway polymorphisms with detailed clinical information and analyses, which provided substantial evidence for the involvement of this pathway in the clinical outcomes of bladder cancer patients, particularly with BCG treatment. There are some limitations in this study. For example, only Caucasians were included. It would be interesting to exam these SNPs in minority populations. Additionally, sample size is not particularly large, although power calculation showed that our analysis had sufficient power (>80%) to detect the main effects analyzed. Although our data are largely internally validated, future replication studies in independent populations are needed to validate some of the results and to translate the findings to clinical trials.

## Supporting Information

Table S1 **Demographic and clinical variables for 421 NMIBC patients.**
(DOC)

Table S2 **Oxidative stress gene SNPs and recurrence risk in overall NMIBC patients.**
(DOC)

Table S3 **Oxidative stress gene SNPs and recurrence risk in NMIBC patients who received TUR only.**
(DOC)

Table S4 **Oxidative stress gene SNPs and recurrence risk in NMIBC patients who received BCG treatment.**
(DOC)
