# Supplementary material for: Association of Polymorphisms in Oxidative Stress Genes with Clinical Outcomes for Bladder Cancer Treated with Bacillus Calmette-Guérin
Source: PLoS One. 2012 Jun 12;7(6):e38533. doi: 10.1371/journal.pone.0038533 (PMC3373532; doi:10.1371/journal.pone.0038533)
Supplement: Table S1 — Demographic and clinical variables for 421 NMIBC patients. (DOC) [file pone.0038533.s001.doc]

**Supplementary information**

**Table S1.** Demographic and clinical variables for 421 NMIBC patients

| Variables | Recurrence (n=232),  N(%) | No recurrence (n=189),  N(%) | *P*^*^ |  | Progression (n=85),  N(%) | No progression (n=336),  N(%) | *P** |
| --- | --- | --- | --- | --- | --- | --- | --- |
| Age, mean(SD) | 63.1(11.1) | 63.6(11.5) | 0.68 |  | 65.5(9.9) | 62.8(11.6) | 0.05 |
| Gender |  |  | 0.11 |  |  |  | 0.01 |
| Male | 197( 84.9) | 149( 78.8) |  |  | 78(91.8) | 268(79.8) |  |
| Female | 35( 15.1) | 40( 21.2) |  |  | 7(8.2) | 68(20.2) |  |
|  |  |  |  |  |  |  |  |
| Smoking status |  |  | 0.69 |  |  |  | 0.96 |
| Never | 64( 27.6) | 55( 29.1) |  |  | 23(27.1) | 96(28.6) |  |
| Former | 120( 51.7) | 90( 47.6) |  |  | 43(50.6) | 167(49.7) |  |
| Current | 48( 20.7) | 44( 23.3) |  |  | 19(22.4) | 73(21.7) |  |
|  |  |  |  |  |  |  |  |
| Stage |  |  | 0.13 |  |  |  | <0.01 |
| Ta | 107(46.5) | 85(45.0) |  |  | 24(28.6) | 168(50.1) |  |
| Tis | 17(7.4) | 6(3.2) |  |  | 8(9.5) | 15(4.5) |  |
| T1 | 106(46.1) | 98(51.9) |  |  | 52(61.9) | 152(45.4) |  |
| Grade |  |  | 0.19 |  |  |  | <0.01 |
| G1 | 5(2.3) | 11(5.9) |  |  | 1(1.3) | 15(4.6) |  |
| G2 | 80(36.9) | 68(36.2) |  |  | 13(16.7) | 135(41.3) |  |
| G3 | 132(60.8) | 109(58.0) |  |  | 64(82.1) | 177(54.1) |  |
| Treatments^#^ |  |  | <0.01 |  |  |  | <0.01 |
| TUR | 99( 42.7) | 45( 23.8) |  |  | 19(22.4) | 125(37.2) |  |
| TUR+iBCG | 91( 39.2) | 30( 15.9) |  |  | 41(48.2) | 80(23.8) |  |
| TUR+iBCG+mBCG | 32( 13.8) | 52( 27.5) |  |  | 16(18.8) | 68(20.2) |  |
| Other | 10( 4.3) | 62( 32.8) |  |  | 9(10.6) | 63(18.8) |  |

*P* values < 0.05 are statistically significant.

**P* values were derived from the χ^2^ test for categorical variables gender, smoking, stage, treatments, and tumor grade, and Student *t*-test was used for the continuous variable age.

^#^ Treatments: TUR subgroup: those who had no further therapy after TUR; TUR+iBCG subgroup: those who received iBCG after TUR; TUR+iBCG+mBCG subgroup: those who further received iBCG and mBCG after TUR treatment; Other subgroup: those who received intravesical chemotherapy but not BCG.
