# Supplementary material for: Association of Polymorphisms in Oxidative Stress Genes with Clinical Outcomes for Bladder Cancer Treated with Bacillus Calmette-Guérin
Source: PLoS One. 2012 Jun 12;7(6):e38533. doi: 10.1371/journal.pone.0038533 (PMC3373532; doi:10.1371/journal.pone.0038533)
Supplement: Table S2 — Oxidative stress gene SNPs and recurrence risk in overall NMIBC patients. (DOC) [file pone.0038533.s002.doc]

**Table S2.** Oxidative stress gene SNPs and recurrence risk in overall NMIBC patients

|  |  | Recurrence Yes/No | | |  |  |  |  |
| --- | --- | --- | --- | --- | --- | --- | --- | --- |
| SNP | Gene | ww | wv | vv | Best Model^#^ | HR (95% CI)* | *P* | *q* |
| rs4639* | *NEIL2* | 52/62 | 102/93 | 57/33 | RES | 1.90(1.39-2.59) | 6x10^-5^ | 4x10^-3^ |
| rs804276* | *NEIL2* | 61/63 | 96/95 | 54/30 | RES | 1.89(1.37-2.59) | 9x10^-5^ | 4x10^-3^ |
| rs804256* | *NEIL2* | 80/83 | 94/84 | 37/21 | RES | 1.95(1.34-2.84) | 5x10^-4^ | 2x10^-2^ |
| rs1874546* | *NEIL2* | 115/120 | 74/58 | 19/10 | ADD | 1.38(1.12-1.70) | 2x10^-3^ | 6x10^-2^ |
| rs804267* | *NEIL2* | 111/76 | 82/84 | 18/28 | RES | 0.75(0.46-1.22) | 3x10^-3^ | 6x10^-2^ |
| rs4135054* | *TDG* | 157/150 | 52/37 | 2/1 | DOM | 1.59(1.15-2.20) | 5x10^-3^ | 8x10^-2^ |
| rs8191604 | *NEIL2* | 123/90 | 70/85 | 18/13 | DOM | 0.69(0.52-0.91) | 8x10^-3^ | - |
| rs10175368 | *CYP1B1* | 101/99 | 82/76 | 28/13 | RES | 1.73(1.15-2.60) | 9x10^-3^ | - |
| rs9332197 | *CYP2C9* | 186/171 | 25/17 | 0/0 | DOM | 1.73(1.13-2.66) | 0.01 | - |
| rs352507 | *SIRT6* | 98/101 | 91/64 | 22/23 | DOM | 1.43(1.08-1.91) | 0.01 | - |
| rs812498 | *TDG* | 124/110 | 82/65 | 5/13 | RES | 0.32(0.13-0.80) | 0.01 | - |
| rs2645447 | *NEIL2* | 115/116 | 77/63 | 18/9 | ADD | 1.30(1.05-1.61) | 0.01 | - |
| rs1052133 | *OGG1* | 133/117 | 64/67 | 14/4 | RES | 2.00(1.15-3.49) | 0.01 | - |
| rs8191529 | *NEIL2* | 172/161 | 37/25 | 2/2 | DOM | 1.53(1.06-2.19) | 0.02 | - |
| rs11773597 | *CYP3A4* | 173/160 | 36/27 | 2/1 | DOM | 1.54(1.06-2.22) | 0.02 | - |
| rs689457 | *NQO1* | 178/147 | 31/36 | 2/5 | DOM | 0.65(0.45-0.94) | 0.02 | - |
| rs6586711 | *NAT1* | 95/94 | 100/75 | 16/19 | DOM | 1.37(1.03-1.80) | 0.03 | - |
| rs4840584 | *NEIL2* | 190/154 | 20/33 | 0/1 | DOM | 0.60(0.38-0.95) | 0.03 | - |
| rs11111858 | *TDG* | 197/181 | 14/6 | 0/0 | DOM | 1.83(1.04-3.21) | 0.04 | - |
| rs1041740 | *SOD1* | 92/102 | 104/70 | 12/14 | DOM | 1.34(1.02-1.77) | 0.04 | - |
| rs162556 | *CYP1B1* | 68/49 | 106/94 | 37/45 | RES | 0.69(0.48-0.99) | 0.04 | - |
| rs1856908 | *CYP2C9* | 76/82 | 106/80 | 29/26 | DOM | 1.34(1.01-1.79) | 0.04 | - |
| rs2173962 | *SOD1* | 184/174 | 26/13 | 1/1 | DOM | 1.53(1.01-2.33) | 0.04 | - |
| rs4135064 | *TDG* | 171/157 | 38/31 | 2/0 | DOM | 1.44(1.01-2.06) | 0.05 | - |
| rs1866074 | *TDG* | 46/50 | 128/95 | 37/43 | DOM | 1.40(1.00-1.96) | 0.05 | - |

* SNPs that remained significant after controlling for multiple comparisons by *q* value (FDR < 10%).

^†^ HR: hazard ratio, CI: confidential interval. HR and 95% CI were adjusted by gender, age, smoking status, tumor stage, and tumor grade.

^#^ Best model: the model with smallest *P* value; DOM: dominant model, RES: recessive model, ADD: addictive model.

ww: homozygous wild-type genotype; wv: heterozygous variant genotype; vv: homozygous variant genotype.
