# Supplementary material for: Association of Polymorphisms in Oxidative Stress Genes with Clinical Outcomes for Bladder Cancer Treated with Bacillus Calmette-Guérin
Source: PLoS One. 2012 Jun 12;7(6):e38533. doi: 10.1371/journal.pone.0038533 (PMC3373532; doi:10.1371/journal.pone.0038533)
Supplement: Table S3 — Oxidative stress gene SNPs and recurrence risk in NMIBC patients who received TUR only. (DOC) [file pone.0038533.s003.doc]

**Table S3.** Oxidative stress gene SNPs and recurrence risk in NMIBC patients who received TUR only

|  |  | Recurrence Yes/No | | |  |  |  |
| --- | --- | --- | --- | --- | --- | --- | --- |
| SNP | Gene | ww | wv | vv | Best Model^#^ | HR (95% CI)* | *P* |
| rs7843046 | *NAT1* | 84/45 | 7/0 | 0/0 | DOM | 3.89(1.65-9.18) | 2x10^-3^ |
| rs11773597 | *CYP3A4* | 69/40 | 21/5 | 1/0 | DOM | 2.15(1.29-3.57) | 3x10^-3^ |
| rs1882018 | *TDG* | 58/18 | 30/22 | 3/5 | ADD | 0.58(0.40-0.84) | 4x10^-3^ |
| rs4135054 | *TDG* | 63/36 | 27/9 | 1/0 | DOM | 2.00(1.23-3.24) | 5x10^-3^ |
| rs2070675 | *CYP2E1* | 51/31 | 34/13 | 6/1 | ADD | 1.59(1.13-2.24) | 8x10^-3^ |
| rs4639 | *NEIL2* | 22/14 | 42/23 | 27/8 | RES | 1.86(1.17-2.97) | 8x10^-3^ |
| rs11111858 | *TDG* | 83/44 | 8/1 | 0/0 | DOM | 2.73(1.23-6.03) | 0.01 |
| rs4135113 | *TDG* | 83/44 | 8/1 | 0/0 | DOM | 2.73(1.23-6.03) | 0.01 |
| rs352507 | *SIRT6* | 47/26 | 35/14 | 9/5 | ADD | 1.53(1.09-2.15) | 0.01 |
| rs1866074 | *TDG* | 17/16 | 52/18 | 22/11 | DOM | 1.99(1.14-3.47) | 0.02 |
| rs2410556 | *NAT2* | 71/27 | 19/16 | 1/2 | DOM | 0.55(0.33-0.91) | 0.02 |
| rs2758329 | *SOD2* | 12/16 | 54/23 | 25/6 | DOM | 2.10(1.12-3.91) | 0.02 |
| rs2758331 | *SOD2* | 26/6 | 53/23 | 12/16 | RES | 0.48(0.26-0.89) | 0.02 |
| rs4880 | *SOD2* | 15/17 | 52/22 | 24/6 | DOM | 1.92(1.09-3.37) | 0.02 |
| rs2758346 | *SOD2* | 15/17 | 52/22 | 24/6 | DOM | 1.92(1.09-3.37) | 0.02 |
| rs3135967 | *LIG3* | 17/14 | 50/25 | 20/6 | ADD | 1.45(1.05-2.02) | 0.03 |
| rs10888150 | *NAT1* | 28/18 | 46/20 | 17/7 | ADD | 1.40(1.04-1.90) | 0.03 |
| rs6586711 | *NAT1* | 36/23 | 42/18 | 13/4 | ADD | 1.42(1.04-1.93) | 0.03 |
| rs804276 | *NEIL2* | 25/13 | 42/24 | 24/8 | RES | 1.71(1.05-2.77) | 0.03 |
| rs1565684 | *NAT2* | 21/19 | 51/19 | 19/5 | DOM | 1.75(1.05-2.90) | 0.03 |
| rs172814 | *TDG* | 68/27 | 23/14 | 0/4 | ADD | 0.63(0.42-0.96) | 0.03 |
| rs7829368 | *NAT1* | 38/22 | 36/16 | 17/7 | ADD | 1.36(1.03-1.80) | 0.03 |
| rs812498 | *TDG* | 57/26 | 33/14 | 1/5 | RES | 0.11(0.02-0.83) | 0.03 |
| rs3890995 | *UNG* | 54/38 | 31/6 | 6/1 | ADD | 1.46(1.03-2.08) | 0.04 |
| rs3799204 | *NQO2* | 82/35 | 9/9 | 0/1 | DOM | 0.45(0.21-0.95) | 0.04 |
| rs4149367 | *NQO2* | 82/35 | 9/9 | 0/1 | DOM | 0.45(0.21-0.95) | 0.04 |
| rs1202186 | *ABCB1* | 35/21 | 47/15 | 9/9 | RES | 0.47(0.23-0.96) | 0.04 |
| rs1874546 | *NEIL2* | 48/32 | 37/11 | 6/2 | DOM | 1.55(1.02-2.36) | 0.04 |
| rs162557 | *CYP1B1* | 59/22 | 26/21 | 6/2 | DOM | 0.62(0.40-0.98) | 0.04 |
| rs4986990 | *NAT1* | 84/45 | 7/0 | 0/0 | DOM | 2.55(1.04-6.27) | 0.04 |
| rs7845127 | *NAT1* | 42/23 | 36/16 | 13/6 | DOM | 1.55(1.01-2.36) | 0.04 |
| rs4840584 | *NEIL2* | 85/35 | 5/10 | 0/0 | DOM | 0.39(0.16-0.98) | 0.05 |
| rs2682587 | *XRCC1* | 55/29 | 33/13 | 3/3 | DOM | 1.54(1.00-2.37) | 0.05 |
| rs11407 | *XRCC1* | 55/29 | 33/13 | 3/3 | DOM | 1.54(1.00-2.37) | 0.05 |

* HR: hazard ratio, CI: confidential interval. HR and 95% CI were adjusted by gender, age, smoking status, tumor stage, and tumor grade.

^#^ Best model: the model with smallest *P* value; DOM: dominant model, RES: recessive model, ADD: addictive model.

ww: homozygous wild-type genotype; wv: heterozygous variant genotype; vv: homozygous variant genotype.
