# Supplementary material for: Association of Polymorphisms in Oxidative Stress Genes with Clinical Outcomes for Bladder Cancer Treated with Bacillus Calmette-Guérin
Source: PLoS One. 2012 Jun 12;7(6):e38533. doi: 10.1371/journal.pone.0038533 (PMC3373532; doi:10.1371/journal.pone.0038533)
Supplement: Table S4 — Oxidative stress gene SNPs and recurrence risk in NMIBC patients who received BCG treatment. (DOC) [file pone.0038533.s004.doc]

**Table S4.** Oxidative stress gene SNPs and recurrence risk in NMIBC patients who received BCG treatment

|  |  | Recurrence Yes/No | | |  |  |  |  |
| --- | --- | --- | --- | --- | --- | --- | --- | --- |
| SNP | *Gene* | ww | wv | vv | Best Model^#^ | HR (95% CI)^†^ | *P* | *q* |
| rs804256* | *NEIL2* | 41/36 | 51/42 | 18/4 | RES | 4.58(2.61-8.02) | 1x10^-7^ | 1x10^-5^ |
| rs804276* | *NEIL2* | 33/31 | 47/41 | 30/10 | RES | 2.71(1.75-4.20) | 9x10^-6^ | 6x10^-4^ |
| rs4639* | *NEIL2* | 28/28 | 52/44 | 30/10 | RES | 2.60(1.68-4.03) | 2x10^-5^ | 8x10^-4^ |
| rs2173962* | *SOD1* | 93/81 | 16/1 | 1/0 | DOM | 2.45(1.42-4.23) | 1x10^-3^ | 4x10^-2^ |
| rs804267* | *NEIL2* | 61/32 | 39/37 | 10/13 | DOM | 0.53(0.36-0.78) | 1x10^-3^ | 4x10^-2^ |
| rs8191604* | *NEIL2* | 73/40 | 31/39 | 6/3 | DOM | 0.54(0.36-0.81) | 3x10^-3^ | 7x10^-2^ |
| rs9332197 | *CYP2C9* | 96/78 | 14/4 | 0/0 | DOM | 2.35(1.28-4.30) | 6x10^-3^ | - |
| rs2645447 | *NEIL2* | 63/51 | 34/28 | 12/3 | RES | 2.38(1.28-4.45) | 6x10^-3^ | - |
| rs4921580 | *NAT1* | 72/63 | 32/10 | 1/4 | DOM | 1.84(1.19-2.86) | 7x10^-3^ | - |
| rs1874546 | *NEIL2* | 61/52 | 33/24 | 13/6 | RES | 2.29(1.25-4.18) | 7x10^-3^ | - |
| rs1013358 | *MPG* | 75/68 | 33/14 | 2/0 | DOM | 1.73(1.14-2.62) | 1x10^-2^ | - |
| rs12863638 | *PARP4* | 64/45 | 38/34 | 7/3 | RES | 2.95(1.29-6.72) | 1x10^-2^ | - |
| rs2010628 | *NEIL2* | 80/47 | 22/31 | 8/4 | DOM | 0.59(0.38-0.90) | 1x10^-2^ | - |
| rs17327624 | *ABCB1* | 64/54 | 39/27 | 7/1 | RES | 2.69(1.20-6.03) | 0.02 | - |
| rs1866074 | *TDG* | 28/19 | 68/43 | 14/20 | RES | 0.49(0.27-0.88) | 0.02 | - |
| rs10888150 | *NAT1* | 43/29 | 54/32 | 13/21 | RES | 0.49(0.27-0.89) | 0.02 | - |
| rs3219476 | *MUTYH* | 52/31 | 47/36 | 11/15 | ADD | 0.72(0.55-0.96) | 0.02 | - |
| rs352500 | *SIRT6* | 25/24 | 64/35 | 21/23 | RES | 0.56(0.34-0.92) | 0.02 | - |
| rs4986993 | *NAT1* | 67/44 | 39/29 | 4/9 | RES | 0.33(0.12-0.89) | 0.03 | - |
| rs15561 | *NAT1* | 67/44 | 38/29 | 4/9 | RES | 0.33(0.12-0.89) | 0.03 | - |
| rs7787082 | *ABCB1* | 88/55 | 17/23 | 2/3 | DOM | 0.57(0.34-0.95) | 0.03 | - |
| rs1052133 | *OGG1* | 62/49 | 40/31 | 8/2 | RES | 2.26(1.07-4.80) | 0.03 | - |
| rs125701 | *OGG1* | 76/59 | 31/17 | 3/6 | RES | 0.28(0.09-0.90) | 0.03 | - |
| rs8191529 | *NEIL2* | 85/71 | 23/11 | 2/0 | DOM | 1.65(1.03-2.64) | 0.04 | - |
| rs689457 | *NQO1* | 91/57 | 18/22 | 1/3 | DOM | 0.59(0.36-0.97) | 0.04 | - |
| rs4840583 | *NEIL2* | 43/24 | 44/43 | 23/14 | DOM | 0.67(0.45-0.98) | 0.04 | - |
| rs3219487 | *MUTYH* | 92/52 | 18/22 | 0/1 | DOM | 0.59(0.35-0.99) | 0.04 | - |
| rs8191534 | *NEIL2* | 76/47 | 26/29 | 8/4 | DOM | 0.66(0.43-0.99) | 0.04 | - |

* SNPs that remained significant after controlling for multiple comparisons by *q* value (FDR < 10%).

^†^ Adjusted for age, gender, smoking status, tumor stage, and tumor grade.

^#^ Best model: the model with smallest *P* value; DOM: dominant model, RES: recessive model, ADD: addictive model.

ww: homozygous wild-type genotype; wv: heterozygous variant genotype; vv: homozygous variant genotype.
